# Supplementary material for: Latent leprosy infection identified by dual RLEP and anti-PGL-I positivity: Implications for new control strategies
Source: PLoS One. 2021 May 13;16(5):e0251631. doi: 10.1371/journal.pone.0251631 (PMC8118453; doi:10.1371/journal.pone.0251631)

**S1 Fig.** Frequency of anti-PGL-I positivity in new cases, post-treated cases, HHC and HEC. A) Positivity versus negativity in anti-PGL-I titers with a similar percentage of positives observed in newly diagnosed cases, post-treated cases, and household contacts, while those in the HEC group were negative or weakly above the cut-off. B) Anti-PGL-I optical density (O.D.) for all individuals were plotted for each group with the median O.D. indicated by the solid horizontal line. The significant *p* value differences between groups are shown.


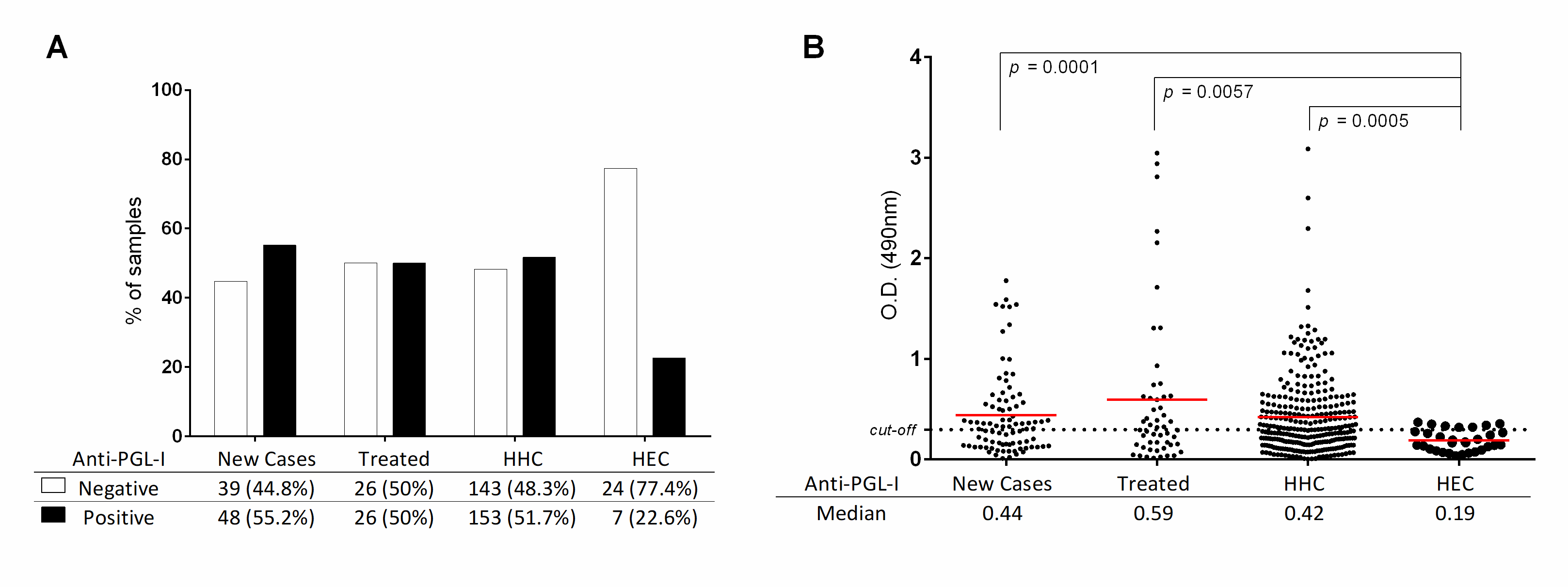

Supplement: S1 Fig — A) Positivity versus negativity in anti-PGL-I titers with a similar percentage of positives observed in newly diagnosed cases, post-treated cases, and household contacts, while those in the HEC group were negative or weakly above the cut-off. B) Anti-PGL-I optical density (O.D.) for all individuals were plotted for each group with the median O.D. indicated by the solid horizontal line. The significant p value differences between groups are shown. (DOC) [file pone.0251631.s001.doc]
