# Supplementary material for: Latent leprosy infection identified by dual RLEP and anti-PGL-I positivity: Implications for new control strategies
Source: PLoS One. 2021 May 13;16(5):e0251631. doi: 10.1371/journal.pone.0251631 (PMC8118453; doi:10.1371/journal.pone.0251631)
Supplement: S2 Fig — A) RLEP positivity within each of the four groups examined based on PCR of earlobe SSS. New cases were 83.9% positive, treated cases were 44.2% positive, HHC were 27.4% positive whereas no HEC were positive (0%). B) anti-PGL-I titer was plotted for each individual based on being RLEP positive or negative within each group. Solid line indicates the median O.D. for each group. There was no significant difference between the median anti-PGL-I titer when positive and negative RLEP groups were compared between any of the two patient and HHC groups. (DOC) [file pone.0251631.s002.doc]

**S2 Fig.** Analysis of RLEP and PGL-I titer for new cases, post-treated cases, HHC and HEC. A) RLEP positivity within each of the four groups examined based on PCR of earlobe SSS. New cases were 83.9% positive, treated cases were 44.2% positive, HHC were 27.4% positive whereas no HEC were positive (0%). B) anti-PGL-I titer was plotted for each individual based on being RLEP positive or negative within each group. Solid line indicates the median O.D. for each group. There was no significant difference between the median anti-PGL-I titer when positive and negative RLEP groups were compared between any of the two patient and HHC groups.

**
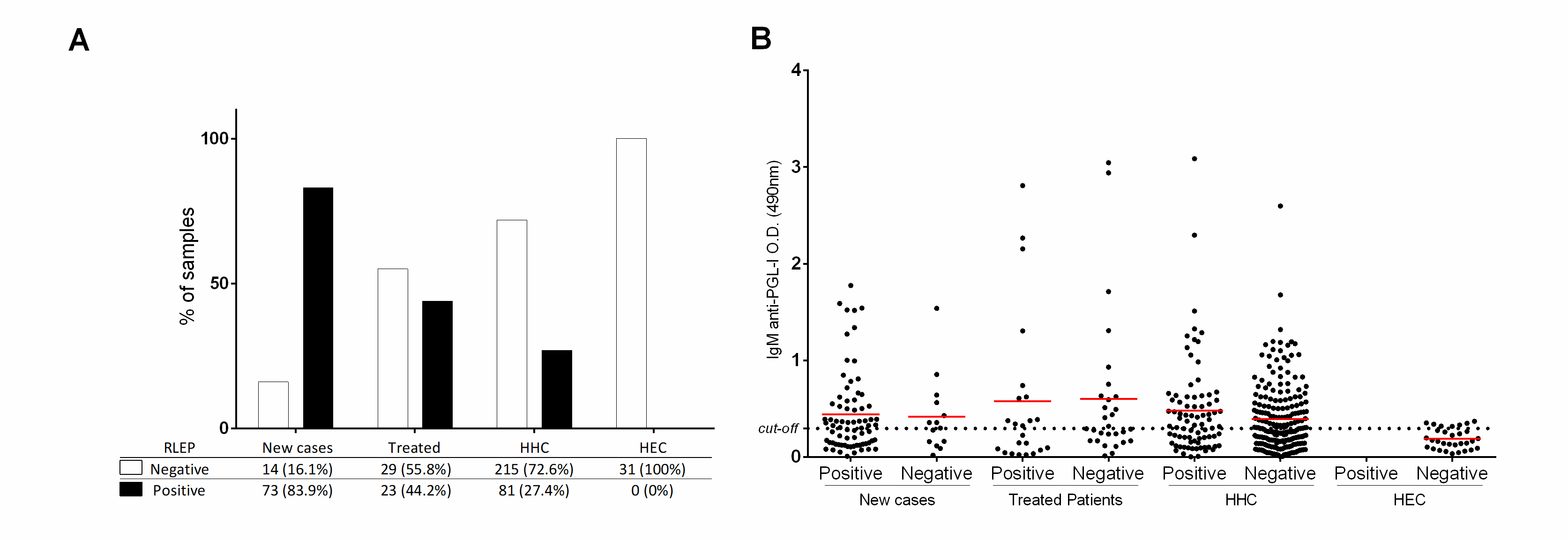
**
