# Supplementary material for: Latent leprosy infection identified by dual RLEP and anti-PGL-I positivity: Implications for new control strategies
Source: PLoS One. 2021 May 13;16(5):e0251631. doi: 10.1371/journal.pone.0251631 (PMC8118453; doi:10.1371/journal.pone.0251631)
Supplement: S1 Table — Characteristics of newly diagnosed leprosy patients, treated leprosy patients, healthy household contacts (HHC) and healthy endemic controls (HEC) from the seven cities surveyed. (DOC) [file pone.0251631.s003.doc]

**S1 Table.** Operational classification and number of subjects per group and municipality. Characteristics of newly diagnosed leprosy patients, treated leprosy patients, healthy household contacts (HHC) and healthy endemic controls (HEC) from the seven cities surveyed.

|  | | New leprosy cases | | | Treated leprosy patients | | | | HHC | HEC |
| --- | --- | --- | --- | --- | --- | --- | --- | --- | --- | --- |
| City | PB | % | MB | % | PB | % | MB | % |  |  |
| Acará | 4 | 50.0 | 4 | 50.0 | 1 | 10.0 | 9 | 90.0 | 66 | - |
| Breves | 1 | 20.0 | 4 | 80.0 | 1 | 100.0 | - | - | 18 | - |
| Castanhal | - | - | - | - | 1 | 20.0 | 4 | 80.0 | 26 | - |
| Belém/Mosqueiro | 6 | 14.6 | 35 | 85.4 | - | - | - | - | 74 | 31 |
| Redenção | 1 | 25.0 | 3 | 75.0 | 2 | 20.0 | 8 | 80.0 | 24 | - |
| Santarém | 4 | 16.7 | 20 | 83.3 | 6 | 37.5 | 10 | 62.5 | 52 | - |
| Senador José Porfirio | - | - | 5 | 100.0 | 3 | 30.0 | 7 | 70.0 | 36 | - |
| Total | 16 | 18.4 | 71 | 81.6 | 14 | 26.9 | 38 | 73.1 | 296 | 31 |
