# Supplementary material for: Latent leprosy infection identified by dual RLEP and anti-PGL-I positivity: Implications for new control strategies
Source: PLoS One. 2021 May 13;16(5):e0251631. doi: 10.1371/journal.pone.0251631 (PMC8118453; doi:10.1371/journal.pone.0251631)
Supplement: S2 Table — Double positive (PGL-I+/RLEP+), single positive (PGL-I+/RLEP- or PGL-I-/RLEP+) and double negative (PGL-I-/RLEP-) were calculated for each of the four groups. The numbers of PB and MB cases are shown for the new case and treated case groups. (DOCX) [file pone.0251631.s004.docx]

**S2 Table.** Correlation of RLEP and anti-PGL-I titer within each group. Double positive (PGL-I+/RLEP+), single positive (PGL-I+/RLEP- or PGL-I-/RLEP+) and double negative (PGL-I-/RLEP-) were calculated for each of the four groups. The numbers of PB and MB cases are shown for the new case and treated case groups.

|  | PGL+  /RLEP+ | | PB | | MB | | PGL-/RLEP+ | | PB | | MB | | PGL+  /RLEP- | | PB | | MB | | PGL-/RLEP- | | PB | | MB | |
| --- | --- | --- | --- | --- | --- | --- | --- | --- | --- | --- | --- | --- | --- | --- | --- | --- | --- | --- | --- | --- | --- | --- | --- | --- |
|  | n | % | n | % | n | % | n | % | n | % | n | % | n | % | n | % | n | % | n | % | n | % | n | % |
| New cases (n = 87) | 40 | 46.0 | 4 | 10 | 36 | 90 | 33 | 37.9 | 7 | 21.2 | 26 | 78.8 | 8 | 9.2 | 2 | 25 | 6 | 75 | 6 | 6.9 | 3 | 50 | 3 | 50 |
| Treated  (n = 52) | 12 | 23.1 | 4 | 33.3 | 8 | 66.7 | 11 | 21.2 | 4 | 36.4 | 7 | 63.6 | 14 | 26.9 | 2 | 14.3 | 12 | 85.7 | 15 | 28.8 | 5 | 33.3 | 10 | 66.7 |
| HHC  (n = 296) | 46 | 15.5 |  |  |  |  | 35 | 11.8 |  |  |  |  | 107 | 36.1 |  |  |  |  | 108 | 36.5 |  |  |  |  |
| HEC  (n = 31) | 0 | 0% |  |  |  |  | 0 | 0% |  |  |  |  | 7 | 22.6 |  |  |  |  | 24 | 77.4 |  |  |  |  |
