# Supplementary material for: Latent leprosy infection identified by dual RLEP and anti-PGL-I positivity: Implications for new control strategies
Source: PLoS One. 2021 May 13;16(5):e0251631. doi: 10.1371/journal.pone.0251631 (PMC8118453; doi:10.1371/journal.pone.0251631)
Supplement: S3 Table — (DOCX) [file pone.0251631.s005.docx]

**S3 Table.** Demographic information of the four groups studied (new leprosy cases, treated patients, HHC and HEC) including household density (average number of people living in the house), type of water used for drinking and cooking, income level, education level, receiving governmental support, median age and range, ratio of number of males to females, incidence of food deprivation and living in an urban area.

|  | **Number of people per house** | **Type of water used**  **for drinking/cooking** | **Salary** | **Education (highest grade)** | **Receive governmental support**  **(%)** | **Median age (range)** | **Sex**  **(ratio M:F)** | **Food deprivation**  **(%)** | **Living in urban area**  **(%)** |
| --- | --- | --- | --- | --- | --- | --- | --- | --- | --- |
| **New cases**  **(n = 87)** | 5.5 | Not treated: 4  Strained water: 39  Chlorinated: 2  Filtered: 36  Mineral water: 6 | ≤ 1 minimum salary: 55  Up to two minimum salary: 21  Up to three minimum salary: 5  Greater than 3MS: 6 | No Education: 66 Elementary School: 11  High school: 9  University education: 1 | 60/87  (69%) | 25  (5-81) | Female: 52  Male: 35  (0.6 : 1) | 18/87  (20.7%) | 81/87  (93.1) |
| **Treated**  **(n = 52)** | 5 | Not treated: 3  Strained water: 21  Chlorinated: 7  Filtered: 12  Mineral water: 5 | ≤ 1 minimum salary: 30  Up to two minimum salary: 13  Up to three minimum salary: 4  Greater than 3MS: 3 | No Education: 30 Elementary School: 8  High school: 8  University education: 6 | 35/52  (67.3%) | 45  (12-87) | Female: 22  Male: 30  (1 : 0.58) | 18/52  (34.6%) | 42/52  (80.8%) |
| **HHC**  **(n = 296)** | 5 | Not treated: 15  Strained water: 147  Chlorinated: 32  Filtered: 70  Mineral water: 32 | ≤ 1 minimum salary: 172  Up to two minimum salary: 81  Up to three minimum salary: 27  Greater than 3MS: 16 | No Education: 106 Elementary School: 127  High school: 52  University education: 11 | 193/296  (65.2%) | 31  (6-79) | Female: 160  Male: 136  (0.46 : 1) | 102/296  (34.4%) | 241/296  (81.4%) |
| **HEC**  **(n = 31)** | 3 | Not treated: 0  Strained water: 0  Chlorinated: 0  Filtered: 2  Mineral water: 29 | ≤ 1 minimum salary: 0  Up to two minimum salary: 1  Up to three minimum salary: 2  Greater than 3MS: 28 | No Education: 0 Elementary School: 0  High school: 0  University education: 31 | 0/31  (0%) | 33  (19-62) | Female: 20  Male: 11  (0.35 : 1) | 0/31  (0%) | 31/31  (100%) |
