# Supplementary material for: Latent leprosy infection identified by dual RLEP and anti-PGL-I positivity: Implications for new control strategies
Source: PLoS One. 2021 May 13;16(5):e0251631. doi: 10.1371/journal.pone.0251631 (PMC8118453; doi:10.1371/journal.pone.0251631)
Supplement: Attachment — Submitted filename: Response to reviewers.pdf [file pone.0251631.s007.pdf]

We thank the reviewers very much for their questions and comments which were very helpful. We have endeavored to answer all of the questions and make the changes and additional clarifying paragraphs to the manuscript based on these suggestions wherever possible, keeping in mind that the reviewers also asked us to shorten the manuscript where possible. Our responses are in blue after each reviewer comment. We hope that the revised version of this manuscript is improved from the original.

PONE-D-20-19814
RLEP and anti-PGL-I double positivity in leprosy patient household contacts represents an important source of transmission in hyperendemic cities in Pará, Brazil.

Revised title: Latent leprosy infection identified by dual RLEP and anti-PGL-I positivity: Implications for new control strategies.

PLOS ONE

Please submit your revised manuscript by **Nov 4, 2020** 11:59PM. Please upload your review as an attachment if it exceeds 20,000 characters

Reviewer #1:

Dear authors,

Please make your manuscript shorter and focus mainly on your findings
Thank you for these suggestions, we will endeavor to make changes wherever possible to achieve this.

line 412 "Prophylactic treatment of this high risk group and their HHC would likely be an effective strategy." what did you mean from prophylactic treatment? MDT or SDR?
This is a very important question that we can address in this response, but we will not debate this in our manuscript because it would be too long and is tangential to our study. Studies to examine the efficacy of the broad use of SDR under the LPEP (Leprosy Post Exposure Prophylaxis) trials have been ongoing since 2015, having been implemented alongside national leprosy programs in India, Indonesia, Myanmar, Nepal, Sri Lanka, Tanzania, Brazil and Cambodia. To date, they have not published any data on the efficacy of widespread use of SDR. There has been much debate among leprosy clinicians and academics as to whether SDR is a cost-effective intervention and there are concerns that a single dose of rifampin is not sufficient to protect against developing multibacillary (MB) disease.[1] Others are concerned about SDR potentially increasing drug resistance. There may also be ethical problems in telling people that they will be protected since results of the large Chemoprophylaxis of Leprosy (COLEP) trial in Bangladesh found that SDR only protects some people from some types of leprosy (not MB disease) and only for up to 2 years.[1] We include a new paragraph in the Discussion to address this.

1. Lockwood DNJ, Krishnamurthy P, Kumar B, Penna G. 2018. Single-dose rifampicin chemoprophylaxis protects those who need it least and is not a cost-effective intervention. PLoS Negl Trop Dis 12: e0006403. doi.org/10.1371/journal.pntd.0006403

Reviewer #2:

The design and research of the paper are highly operable. It has certain value for the diagnosis and prevention of leprosy. The disadvantage is that the sensitivity of the test is low, and the leprosy patients cannot be completely screened.
Thank you for these observations. We agree that the sensitivity of the anti-PGL-I ELISA is low in the newly diagnosed patient group (55%), but the majority of these patients are categorized as BT (42/87, 48.3%), and since patients in this category have a low BI, anti-PGL-I responses are generally weak or negative compared to

MB patients. Those in the HHC group had a positivity rate slightly less than the new patient group. This is the main reason why we decided to couple anti-PGL-I with RLEP PCR results from earlobe skin smears, as the RLEP positivity in the new patient group was 83.9% (73/87). By detecting the number of double positives in HHC (15.5%) and showing that double positivity represents the largest percentage in newly diagnosed leprosy patients (46%), it suggests to us that this group of HHC has the highest risk of disease progression. Our findings are similar to results in other articles published in the literature, showed a sensitivity of the serological test of 55% in a hyper-endemic population, and 84% for conventional PCR, while the association of both tests reached 88% sensitivity.

The detection sensitivity presented by conventional PCR (88%) to a similar test for tuberculosis approved by the FDA (TB Amplicor-Roche), which presents 79 to 91% sensitivity[1], was superior to staining methods, which shows around 59% sensitivity.[2] We recognize that greater sensitivity is the goal to be achieved, however other detection tools, such as qPCR and ddPCR, are being tested by us to achieve that goal.

1. Yang S and Rothman RE. 2004. PCR-based diagnostics for infectious diseases: uses, limitations, and future applications in acute-care settings. Lancet Infect Dis 4: 337-48.

2. Girma S, Avanzi C, Bobosha K, Desta K, Idriss MH, Busso P, et al. 2018. Evaluation of Auramine O staining and conventional PCR for leprosy diagnosis: A comparative cross-sectional study from Ethiopia. PLoS Negl Trop Dis 12(9): e0006706. doi.org/10.1371/journal.pntd.0006706

Reviewer #3:

The overall recommendation to the authors is they should well acquit themselves with the STROBE guidelines and re-write the paper (I'm assuming the study design is cross-sectional). The paper has an overall message they are trying to communicate but it is not supported by the methods, results and the discussion.
Thank you for your suggestions. We will adhere to the STROBE guidelines where possible.

Abstract
The abstract vaguely reports what the problem is, what is known about the problem and what the authors did to solve the problem.
Thank you for your suggestion. Lines 37-45 in the Abstract straightforwardly lay out what the problem is, what is known about the problem and what we did to solve the problem. "In order to more effectively interrupt the chain of transmission, new strategies will be required to detect those with subclinical disease who contribute to spreading disease. To improve the ability to diagnose leprosy earlier in asymptomatic infected individuals, we examined the combined use of two well-known biomarkers of *M. leprae* infection, namely the presence of *M. leprae* DNA by PCR from earlobe slit skin smears (SSS) and positive serum antibody to the *M. leprae*-specific Phenolic Glycolipid I antigen (anti-PGL-I) from leprosy patients and household contacts living in seven hyperendemic cities in the northern state of Para, Brazilian Amazon."

However on introducing the bio-markers they should have ideally been written in full. Then abbreviations could be used thereafter (line 40 and 41).
Thank you for your suggestion. The term for earlobe slit skin smears (SSS) is defined in line 40, while the presence of testing *M. leprae* DNA by PCR, these abbreviations should not have to be further explained. The term for the antibody to the M. leprae-specific antigen, Phenolic Glycolipid I (PGL-I) is now first defined in line 43.

Introduction
The introduction overall fairy tries to highlight what the problem is, what the gaps are and what was done about it. However: (Line 118) The Dr. Marcelo Candia Reference Unit in Sanitary Dermatology (UREMC), I don't understand the relevance of this clinic. Does it offer the standard testing; is it the Center of Excellence in Para? More clarity maybe needed for the reader.

Thank you for your suggestion. The Dr. Marcelo Candia Reference Unit in Sanitary Dermatology (UREMC) is the Pará state reference laboratory for the diagnosis and treatment of Hansen's disease and offers specialized multidisciplinary clinical care to its users. UREMC was responsible for 19.9% (489/2,548) of the total leprosy notifications in Pará in 2019. People with suspected signs of leprosy from all over the state are referred to UREMC, some who have been misdiagnosed for years. Several leprosy dermatologists, including Dr. Salgado, have been examining leprosy patients and diagnosing them based on clinical signs and symptoms for over 21 years. Follow-up laboratory tests can include bacilloscopy of skin lesions to determine the BI and/or presence of acid fast bacilli as well as determining the anti-PGL-I titer, both which can be confirmatory in the diagnosis and are used to determine the length of the treatment regimen, MDT-PB or MDT-MB. We have modified the text to reflect the importance of this leprosy reference laboratory in the care and treatment of leprosy patients.

Line 144-Line 149 Are actually Methods and Materials in the introduction section. The study design has been highlighted as well as the study description. I think this should be moved to the methods section.
According to the STROBE checklist for cross-sectional studies (von Elm et al. Lancet, 2007), in the Introduction item #2 for Background/rationale states “Explain the scientific background and rationale for the investigation being reported.” These sentences are critically important to establish the rationale for investigating these two particular biomarkers in leprosy patients, healthy household contacts and healthy endemic control subjects that we examined.

Line 149-line 153 this is actually the discussion section in the introduction. This part should be moved to the discussion section.
Again, according to the STROBE checklist for cross-sectional studies, in the Introduction item #3 for Objectives states “State specific objectives, including any prespecified hypothesis”. These sentences are critically important so that the specific objectives are made clear and a hypothesis about the potential risks for individuals who are double positive for both biomarkers is also stated clearly.

Methods
Overall, the methods are poorly presented and hence the study cannot be reproduced. We don’t know what study design was used. A prospective study design was mooted in the introduction but it is not anywhere else. I advise the authors to look up the STROBE guidelines by Von Elm et al.
This the study is about probable diagnostic tests, the authors should tell us if the tests were done in series or in parallel since the results change depending on what is done.
There are some result tables within the methods section.
Thank you for your suggestion. According to the STROBE checklist for cross-sectional in series studies, we have included the following items in the Materials and Methods section and made some selective modifications in the text on the Study Design, Setting, Participants, Variables, Data measurements, Study size, Quantitative variables and Statistical methods. We have now added a new paragraph “Sampling design and methods”, lines 180-202, to further clarify how patients and household contacts were identified and sampled. As far as reproducibility, the ELISA anti-PGL-I assay and RLEP PCR described have been in use by us for over 12 years and both of these tests have been used by leprosy investigators all over the world, they are well-established and easily reproducible in well equipped laboratories. Based on item #10 for Study size the STROBE checklist states “Explain how the study size was arrived at”, Table 1 shows the number of individuals in each group (new leprosy patients, treated leprosy patients, healthy household contacts and healthy endemic controls) and from what cities they came from to answer this question. We have moved Table 1 to the Results section.

Results
I still advise the authors to have a look at the STROBE guidelines. However, it is important to add the socio-demographics of the study subjects unless this is a secondary analysis. The socio-demographics will help us with generalization and also perspective.
The author should use the statistical methods mentioned in the results section to find out if they are significant. Currently all we have are the proportions. We are not sure if they are purely by chance.

Thank you for your suggestion. Sociodemographic aspects are extremely relevant for understanding the epidemiology of leprosy, historically recognized as a disease of poverty[1] and sociodemographic data have previously been explored by other groups for meta-analysis studies[2]. Although this data was not the primary focus of this study, we did collect it from all of the study participants. In previous studies by us, we highlighted potential risk factors that likely lead to higher rates of leprosy in the Amazon region including living in a very high or hyperendemic area for leprosy, poverty, lack of clean water and sanitation in the house, high household density with more than 2 people sleeping per bedroom, poor nutritional status and lack of health care availability.[3,4] Certainly the individuals in the leprosy patient, treated patient and healthy household contact groups are more disadvantaged and likely have multiple risk factors among the above, while those in the healthy endemic group who are mostly university students are better off and live in higher socioeconomic areas of the capital of Belem. We have added Table 3 (see below) that includes this sociodemographic information in the Results and a paragraph in the Discussion.

1. Nery JS, Ramond A, Pescarini JM, Alves A, Strina A, Ichihara MY, Penna MLF, Smeeth L, Rodrigues LC, Barreto ML, Brickley EB, Penna GO. 2019. Socioeconomic determinants of leprosy new case detection in the 100 Million Brazilian Cohort: a population-based linkage study. *Lancet Glob Health* 7: e1226-1236.

2. Pescarini JM, Strina A, Nery JS, Skalinski LM, Andrade KVF, Penna MLF, et al. 2018. Socioeconomic risk markers of leprosy in high-burden countries: A systematic review and meta-analysis. *PLoS Negl Trop Dis* 12(7): e0006622. doi.org/10.1371/journal.pntd.0006622

3. Barreto, J. G., D. Bisanzio, L.deS. Guimarães, J. S. Spencer, G. M. Vazquez-Prokopec, U. Kitron, and C. G. Salgado. 2014. Spatial analysis spotlighting early childhood leprosy transmission in a hyperendemic municipality of the Brazilian Amazon region. *PloS Negl. Trop. Dis.* 8(2):e2665. doi: 10.1371/journal.pntd.0002665.

4. Barreto JG, Bisanzio D, Frade MAC, Moraes TMP, Gobbo AR, Guimarães L de S, da Silva MB, Vazquez-Prokopec GM, Spencer JS, Kitron U, Salgado CG. 2015. Spatial epidemiology and serologic cohorts increase the early detection of leprosy. *BMC Inf Dis* 15: 527 doi 10.1186/s12879-015-1254-8.

As for the application of statistical tests:
Lines 257-258: The statistical differences between groups were calculated by Student's t-test
Lines 280-282: When individuals in each group were divided into RLEP positive or negative and examined for anti-PGL-I titer, there were no statistical differences between the median O.D. values for RLEP positive versus negative individuals within the patient or HHC groups (Figure 2B).

Discussion
Overall the discussion is about the bio-markers and leprosy in general. Very little effort is made to discuss the results. Their significance and the recommendations if any, the overall recommendation is hinted all over the paper with no possible justification given from the results.
We have expanded the discussion of the results.

Reviewer #4:
1) This is a good study trying to address the issue of transmission in leprosy.
Thank you for this comment. We feel that there are many unknowns about leprosy transmission that unfortunately cannot be answered because *M. leprae* is not cultivatable.

2) In fact this investigation is trying to look at possible sources of infection in the community and their role in transmission of the disease.
Thank you for this comment. In hyperendemic areas as in all of the cities that we visited in this state, the percentage of people diagnosed by an experienced leprosy dermatologist based on clinical signs and symptoms ranged from 3.4% to 22.3%. These are truly staggering numbers, hundreds of time higher than the nationally

reported new case detection rate (NCDR) reported yearly by the Brazil Ministry of Health database (SINAN), which usually relies on passive detection rates (currently around 1.3 new cases per 10,000 population). We have published these results in numerous articles over the years highlighting that active surveillance by us in cities in the state of Pará generally shows a new case detection rate of around 4% in children and 8% in household contacts in this region.

3) In Table 2 for all parameters the author should give number of MB and PB cases in each group
We have revised Table 2 that shows the breakdown of MB and PB cases for the new case and treated groups for each test combination (double positive, single positive and double negative). MB and PB case categories do not apply for the HHC and HEC groups.

	PGL+/ RLEP+		PB		MB		PGL-/RLEP+		PB		MB		PGL+/RLEP-		PB		MB		PGL-/RLEP-		PB		MB	
	n	%	n	%	n	%	n	%	n	%	n	%	n	%	n	%	n	%	n	%	n	%	n	%
New cases (n = 87)	40	46.0	4	10	36	90	33	37.9	7	21.2	26	78.8	8	9.2	2	25	6	75	6	6.9	3	50	3	50
Treated (n = 52)	12	23.1	4	33.3	8	66.7	11	21.2	4	36.4	7	63.6	14	26.9	2	14.3	12	85.7	15	28.8	5	33.3	10	66.7
HHC (n = 296)	46	15.5					35	11.8					107	36.1					108	36.5				
HEC (n = 31)	0	0%					0	0%					7	22.6					24	77.4				

Table 3. Demographic information of the four groups studied (new leprosy cases, treated patients, healthy household contacts and healthy endemic controls. Highlights of findings: There are overall slightly more females than males except in the treated group, but the ratio does not seem to be overly skewed. Some observations: it is apparent that all of the HEC have a clean source of drinking water (2 filtered, 29 bottled mineral water) while less than half of the people in the other three groups have access to this, possibly leading to more exposure to water borne pathogens such as amoeba and bacteria causing diarrheal disease. Income for HEC is also skewed to 100% having two minimum salaries or more whereas more than half of people in the other three groups have one minimum salary or less, suggesting conditions of poverty. Education levels for HEC are all at university level, while the other three groups have a majority with no education or only primary level schooling. Food deprivation is between 20% (one in 5) to 34% (one in three) for three groups, while HEC do not experience this problem of hunger at all. The number of people per household is around 3 for HEC while at least 5 for the other three groups, indicating overcrowded conditions.

	Number of people per house	Type of water used for drinking/cooking	Salary	Education (highest grade)	Receive governmental support (%)	Median age (range)	Sex (ratio M:F)	Food deprivation (%)	Living in urban area (%)
New cases (n = 87)	5.5	Not treated: 4<br>Strained water: 39<br>Chlorinated: 2<br>Filtered: 36<br>Mineral water: 6	≤ 1 minimum salary: 55<br>Up to two minimum salary: 21<br>Up to three minimum salary: 5<br>Greater than 3MS: 6	No Education: 66<br>Elementary School: 11<br>High school: 9<br>University education: 1	60/87 (69%)	25 (5-81)	Female: 52<br>Male: 35<br>(0.6 : 1)	18/87 (20.7%)	81/87 (93.1)
Treated (n = 52)	5	Not treated: 3<br>Strained water: 21<br>Chlorinated: 7<br>Filtered: 12<br>Mineral water: 5	≤ 1 minimum salary: 30<br>Up to two minimum salary: 13<br>Up to three minimum salary: 4<br>Greater than 3MS: 3	No Education: 30<br>Elementary School: 8<br>High school: 8<br>University education: 6	35/52 (67.3%)	45 (12-87)	Female: 22<br>Male: 30<br>(1 : 0.58)	18/52 (34.6%)	42/52 (80.8%)

HHC (n = 296)	5	Not treated: 15<br>Strained water: 147<br>Chlorinated: 32<br>Filtered: 70<br>Mineral water: 32	≤ 1 minimum salary: 172<br>Up to two minimum salary: 81<br>Up to three minimum salary: 27<br>Greater than 3MS: 16	No Education: 106<br>Elementary School: 127<br>High school: 52<br>University education: 11	193/296 (65.2%)	31 (6-79)	Female: 160<br>Male: 136<br>(0.46 : 1)	102/296 (34.4%)	241/296 (81.4%)
HEC (n = 31)	3	Not treated: 0<br>Strained water: 0<br>Chlorinated:<br>Filtered: 2<br>Mineral water: 29	≤ 1 minimum salary: 0<br>Up to two minimum salary: 1<br>Up to three minimum salary: 2<br>Greater than 3MS: 28	No Education: 0<br>Elementary School: 0<br>High school: 0<br>University education: 31	0/31 (0%)	33 (19-62)	Female: 20<br>Male: 11<br>(0.35 : 1)	0/31 (0%)	31/31 (100%)

4) In HHC it will be interesting to know the type of Index cases whether they are PB or MB
This data was not collected.

5) If skin smear BI available in Index case was there a correlation between high BI positivity and HHC positive
Since most of the work was done in the field, the BI of index cases was not assessed. However, we always do a bacilloscopy on leprosy cases attended at the UREMC reference center, resulting in a high correlation between our clinical definition and the BI.

6) Is there a correlation between smear positivity (If available) and positivity in the two tests performed?
We think there is since double positivity is highest in the new case group (46%) while it is markedly reduced in the treated patient group (23.1%) showing the treatment efficacy of MDT.

7) In HHC was there any other household which had many members who were positive for any of the two tests and the author should mention about number of families which were positive.
The size of each household visited varied considerably, from 3 to 12 and sampling at each house relied on who was at home at the time we visited with some household members away or at work. We cited one family as an example where out of twelve individuals with one index case, six other blood related individuals in this household were diagnosed that day and double positivity in this particular family was 75%. Although this was an extreme finding, there were other families where more than one person was diagnosed in the household that showed high rates of being double positive.

8) In HHC compare the positivity in different age groups and was there any correlation with higher age group?
We now have a new table that shows some of the variables for each of the groups (age, sex, income, household density, availability of clean water, etc.) (Table 3).

9) The author needs to mention about the age groups of subjects and among the cases positive for any test in all groups, was there any child case?
The total population evaluated is made up of 466 individuals, of which 92/466 (19.7%) were under 15 yo. Of the 87 new cases, 38/87 (43.7%) were in children under 15 years old. We have added this to the Results section.

10) There is no legend for the Tables mentioned
All tables have a legend that appears just above the tables.

Lines 237-240: Table 1. Operational classification and number of subjects per group and municipality. Characteristics of newly diagnosed leprosy patients, treated leprosy patients, healthy household contacts (HHC) and healthy endemic controls (HEC) from the seven cities surveyed.

Lines 294-297: Table 2. Correlation of RLEP and anti-PGL-I titer within each group. Double positive (PGL-I+/RLEP+), single positive (PGL-I+/RLEP- or PGL-I-/RLEP+) and double negative (PGL-I-/RLEP-) were calculated for each of the four groups.

11) How many HHC who were doubly positive were followed up if followed up and what was the outcome of that follow up?

We intend to do this in the future as we are getting funding for a project for long-term follow-up of contacts who were clinically healthy, but with positive laboratory results (ELISA and/or PCR). The main limiting factor is the lack of resources available for follow-up visits, which is certainly desirable in studies involving neglected chronic diseases like leprosy. Resources for continued leprosy surveillance projects are needed especially in hyperendemic areas, such as the state of Pará, with a territorial area of 1,248,000 km^2, and the municipalities selected in this project represent each one of the state's macro-regions. Follow-up visits to several of these cities are not possible logistically. For example, Senador José Porfirio is 516 miles away from the capital, Belém, and can only be reached by driving over 18 hours on the Trans-Amazon Highway. Another city, Breves, can only be reached by taking an overnight boat trip by river. In some cities visited we have provided the diagnosis and positivity data to the local health municipality leprosy control coordinators to perform follow-up with these families. Cities that are more easily visited like Acará, Belém and the surrounding metropolitan area (Mosqueiro) and others will be followed up in a future study. We have already published one study and a 2-year follow-up in the city of Castanhal where we found that in a follow-up of 10 individuals who came from anti-PGL-I positive households there would be a 90% chance of diagnosing one new case within 2 years and that individuals in anti-PGL-I positive households had a 2.7-fold higher risk of progressing to disease than those from negative households.[1,2]

1. Barreto JG, Bisanzio D, Guimarães L de S, Spencer JS, Vazquez-Prokopec GM, Kitron U, Salgado CG. 2014. Spatial analysis spotlighting early childhood leprosy transmission in a hyperendemic municipality of the Brazilian Amazon region. *PLoS Negl Trop Dis* 8(2): e2665. doi: 10.1371/journal.pntd.0002665.

2. Barreto JG, Bisanzio D, Frade MAC, Moraes TMP, Gobbo AR, Guimarães L de S, da Silva MB, Vazquez-Prokopec GM, Spencer JS, Kitron U, Salgado CG. 2015. Spatial epidemiology and serologic cohorts increase the early detection of leprosy. *BMC Inf Dis* 15: 527 doi 10.1186/s12879-015-1254-8.

Reviewer #5:

Leprosy is still a public health problem in some countries. In Brazil, the highest prevalence of the disease is observed in North, Northeast and Midwest regions. One important fact regarding the elimination of leprosy is the absence of a gold standard test for diagnosis that results in a significant number of hidden cases. Prophylactic treatment of contacts from multibacillary patients has been evaluated as an effective strategy to control the disease, but the identification of contacts with subclinical infection is important to determine the targets of chemoprophylactic strategy. Here, Silva and colleagues described that contacts that are positive for both RLEP and PGL-1 have latent disease and are at highest risk of progressing to clinical disease. This paper is interesting. However, I have some concerns:

1. Although the experimental design seems correct, authors need to describe the

methodology for analyzing the data. How did they calculate sensitivity, specificity and accuracy? Please include in the methodology section.

Thank you for this question. Accuracy is the test ability to identify correctly subjects with leprosy (true positive) and healthy endemic controls (true negative). Sensitivity is the test ability to identify correctly subjects with leprosy (true positive). Specificity is the test ability to identify correctly subjects without leprosy, named as healthy endemic controls (true negative). All concepts described above were based on well-established terms of statistics and contextualized to leprosy disease[1,2]. Sensitivity, accuracy, and specificity were determined by the exact method of Clopper and Pearson[3] using the GraphPad prism 6 (GraphPad Software, California, USA).

1. Gurung P, Gomes CM, Vernal S, Leeflang MMG. 2019. Diagnostic accuracy of tests for leprosy: a systematic review and meta-analysis. Clin Microbiol Infect 25: 1315-1327.

2. Vogels CBF, Brito AF, Wyllie AL et al. 2020. Analytical sensitivity and efficiency comparisons of SARS-CoV-2 RT-qPCR primer-probe sets. Nat Microbiol doi.org/10.1038/s41564-020-0761-6

3. Clopper C and Pearson ES. 1934. The use of confidence or fiducial limits illustrated in the case of the binomial. Biometrika 26: 404-413. doi:10.1093/biomet/26.4.404

2. The "Discussion" is rather long and sometimes confusing. It contains common information that is not directly related to the specific topic of this study.

We will try our best to limit the Discussion directly to our results although we were asked to include additional data in Table 3 with discussion.

3. Several studies have reported that PGL-I is a marker of exposure, but not necessarily of infection. It is not clear in the discussion what is the hypothesis for the different profiles observed in the contacts. For example, PGL-I+/RLEP+ are the latent patients. But what is the hypothesis for PGL-I-/RLEP+? Please discuss it.

These are excellent questions. We would argue that in order to induce a positive anti-PGL-I response there must be an active infection. There is consensus and support in the literature that “the presence of circulating anti-PGL-I among healthy contacts was considered to indicate a subclinical infection”.[1] However, not everyone who is infected has a positive antibody response, e.g. only around 20-40% of diagnosed PB patients have a positive titer because their bacillary load is low. Studies indicate that having a positive anti-PGL-I titer increases the risk of succumbing to leprosy by around 6-fold.[2] Our hypothesis is that those individuals who are PGL-I-/RLEP+ are also subclinical, they just do not have a sufficient bacillary load to induce an anti-PGL-I response. We are attempting to use quantitative RT-PCR to correlate the bacillary load in earlobe SSS with the anti-PGL-I response. We have added a paragraph into the Discussion to clarify this:

The four different possible combinations of ELISA/PCR results can be cautiously interpreted in several ways. We propose that those individuals without clinical signs and symptoms of leprosy who are PGL-I+/RLEP+ have latent leprosy infection, allowing permissive growth to allow infection of *M. leprae* in the earlobe and spread to other sites in the skin and an antibody response. These individuals most resemble newly diagnosed patients, the majority of whom are double positive, and thus are at the greatest risk of progressing to disease. Individuals who are PGL-I+/RLEP- are infected

but their functional cell mediated immune response has limited bacterial infection in the earlobe, which can evolve to a cure or can progress to paucibacillary disease. PGL-I-/RLEP+ individuals are also infected but the bacillary load has not increased to the point that induces an anti-PGL-I response. These individuals could either control the bacilli or progress to disease if the cell mediated response allows permissive growth and spread. Individuals who are double negative, PGL-I-/RLEP-, may not have been exposed to enough of a bacterial load to infect them or were more resistant to infection. These results could change over time depending on continued exposure to an untreated index case or other factors influencing a robust cell mediated immune response (co-infections, poor nutritional status).

1. Araújo S, Lobato J, Reis E de M, Souza DOB, Goncalves MA, Costa AV, Goulart LR, Goulart IMB. 2012. Unveiling healthy carriers and subclinical infections among household contacts of leprosy patients who play potential roles in the disease chain of transmission. Mem Inst Oswaldo Cruz 107 (Suppl I): 55-59.

2. Goulart IMB, Souza DOB, Marques CR, Pimenta VL, Goncalves MA, Goulart LR. 2008. Risk and protective factors for leprosy development determined by epridemiological surveillance of household contacts. Clin Vacc Immunol 15: 101-105.
