# Supplementary material for: Latent leprosy infection identified by dual RLEP and anti-PGL-I positivity: Implications for new control strategies
Source: PLoS One. 2021 May 13;16(5):e0251631. doi: 10.1371/journal.pone.0251631 (PMC8118453; doi:10.1371/journal.pone.0251631)
Supplement: Attachment — Submitted filename: Comments to the authors.docx [file pone.0251631.s006.docx]

**Comments to the authors.**

The overall recommendation to the authors is they should well acquit themselves with the STROBE guidelines and re-write the paper (I’m assuming the study design is cross-sectional). The paper has an overall message they are trying to communicate but it is not supported by the methods, results and the discussion.

**Abstract**

The abstract vaguely reports what the problem is, what is known about the problem and what the authors did to solve the problem.

However on introducing the bio-markers they should have ideally been written in full. Then abbreviations could be used thereafter (line 40 and 41).

**Introduction**

The introduction overall fairy tries to highlight what the problem is, what the gaps are and what was done about it. However:

(Line 118) The Dr. Marcelo Candia Reference Unit in Sanitary Dermatology (UREMC), I don’t understand the relevance of this clinic. Does it offer the standard testing; is it the Center of Excellence in Para? More clarity maybe needed for the reader.

Line 144-Line 149 Are actually Methods and Materials in the introduction section. The study design has been highlighted as well as the study description. I think this should be moved to the methods section.

Line 149-line 153 this is actually the discussion section in the introduction. This part should be moved to the discussion section.

**Methods**

Overall, the methods are poorly presented and hence the study cannot be reproduced. We don’t know what study design was used. A prospective study design was mooted in the introduction but it is not anywhere else. I advise the authors to look up the STROBE guidelines by Von Elm et al.

This the study is about probable diagnostic tests, the authors should tell us if the tests were done in series or in parallel since the results change depending on what is done.

There are some result tables within the methods section.

**Results**

I still advise the authors to have a look at the STROBE guidelines. However, it is important to add the socio-demographics of the study subjects unless this is a secondary analysis. The socio-demographics will help us with generalization and also perspective.

The author should use the statistical methods mentioned in the results section to find out if they are significant. Currently all we have are the proportions. We are not sure if they are purely by chance.

**Discussion**

Overall the discussion is about the bio-markers and leprosy in general. Very little effort is made to discuss the results. Their significance and the recommendations if any, the overall recommendation is hinted all over the paper with no possible justification given from the results.
