# Supplementary material for: Latent leprosy infection identified by dual RLEP and anti-PGL-I positivity: Implications for new control strategies
Source: PLoS One. 2021 May 13;16(5):e0251631. doi: 10.1371/journal.pone.0251631 (PMC8118453; doi:10.1371/journal.pone.0251631)
Supplement: Attachment — Submitted filename: Response to Reviewers.doc [file pone.0251631.s009.doc]

We again thank the reviewers and the editor very much for their additional valuable input and suggestions which were very helpful. We have endeavored to answer all of the questions and make the changes to the manuscript based on these suggestions wherever possible. Our point-by-point responses are in blue after each reviewer comment and describe the changes made to the manuscript in response to their comments. We hope that the second revised version of this manuscript addresses the remaining issues.

PONE-D-20-19814 R1

Title: Latent leprosy infection identified by dual RLEP and anti-PGL-I positivity: Implications for new control strategies.

PLOS ONE

By considering the lengthy review process and considerable improvement in the revised manuscript, we would like to give the authors a final chance to fix the issues. If you can address the points raised by editors and reviewers, we would encourage you to submit a revised manuscript. Once we have received your revised manuscript, a decision will be made, which we expect by 22 February 2021.

**Comments from editors**

The quality of the English used throughout your manuscript does not currently meet our minimum requirements, as there are substantial incorrect sentence constructions and grammatical errors throughout, obscuring the message the authors want to convey. After discussing the paper further, the editors felt that the manuscript need significant changes, mostly the sentences rephrasing. We recommend consulting native speakers.

The quality of the English used throughout your manuscript is not acceptable for publication. Many sentences are of 4 to 5 lines; sometimes, it's impossible to understand what authors want to convey. I suggest to break the long sentence and make it concise and clear. The first sentence of the abstract itself confusing. I feel like reading google translated texts. Also, please check the word "Positivity" in the title is suitable over there?

This is the third revision of this manuscript, once to PLoS Neglected Tropical Diseases in 2019 (7 months under review) and twice to PLoS ONE. The original submission and each revision of the text was handled mainly by Dr. Spencer, who is a native English speaker. He has gone through the text to re-write, shorten or break up any run-on sentences for better clarity and readability.

The first sentence of the Abstract has been re-written.

We believe that the word “positivity”, referring to the fact that both RLEP and anti-PGL-I are positive, is suitable.

**Comments from Reviewer #3**

The introduction does clearly explain the problem, but it could be summarized. The flow is good till line 109, however after that what was done should have been summarized.

We have significantly cut, revised and summarized the Introduction.

I understand the importance of UREMC in confirmation of Leprosy disease however, it is just a referral site for the region and the authors should state likewise. However, if the authors must include it in the paper, then they should shift it to the methods section, and then give details about it and what role it plays including the staff who they are and what they do.

The importance of UREMC was expanded in the last revision at the request of this reviewer and is only important in the context of illustrating the full range of laboratory tests (determination of the BI, confirming acid fast bacilli in skin smears, histopathology of skin lesions and anti-PGL-I titer by ELISA) used at this referral site to confirm the correct diagnosis for leprosy patients newly diagnosed at this facility. Since the subjects in this study provided blood and earlobe SSS in the field that were later analyzed in our laboratory, we agree with the reviewer that mentioning and providing details about the UREMC leprosy reference center can be removed for the sake of brevity.

Line 145-to Line 154 is actually Methods and Materials and the results in the introduction section. Despite the authors attempt to highlight that they are giving a rationale for the investigation. They actually stated the study design and what they found. If they were tempting to give the hypothesis, there is need for re-wording
“We show that the majority of newly diagnosed leprosy cases with clinical symptoms are positive for both of these biomarkers of infection suggesting the possibility that household contacts who are also double positive may have latent disease and should be carefully monitored through follow-up examinations.”

We have eliminated this sentence from this section.

It may be important to state the sampling methods used to get the evaluation areas. The authors do not that it was divided into Rural and Urban. But there is no other mention of any sampling technique and why it was chosen.

The municipalities selected, as mentioned in “Study area”, were “To include a broad representation of individuals from cities of various sizes, we selected seven municipalities that came from the six mesoregions in this state.” These sites provided a diverse set of municipalities and study subjects from very small towns with less than 12,000 population to the capital city with a population of 2.3M.

It may be important to find out how the authors arrived at 466 individuals with 87 newly infected, 52 former patients, 296 household contacts and 31 healthy endemic controls. Was this also random or it was the available data?

The first three groups were a set of available data. The 31 healthy endemic controls were added in response to the request from a reviewer that we needed to add this control group when the manuscript was submitted to PLoS Neglected Tropical Diseases the first time in June, 2019.

There is completely no mention of how the data was collected or stored prior to being analyzed

Thank you for mentioning this. We assume that you mean “biological samples” for “data”. We have added two sentences regarding the processing and storage of the serum and earlobe SSS in “Study Design and Methods”. “Blood was processed by centrifugation at each site to yield serum that was frozen and transported to the laboratory for analysis by ELISA. Earlobe SSS samples were placed in microcentrifuge tubes containing 70% ethanol and transported to the laboratory for DNA extraction and analysis by PCR.”

Line 255 to 256, in the results section we expect to get the results got from the use of the t-test not what statistical test was used.

We have changed this sentence to state that the p values were calculated to show significant differences between groups.

This may seem like a contradiction on my last review, but I think there is need summarize the key finding of the study and discuss only those.

Please understand that there were 4 reviewers who each requested that we make changes and additions to the Discussion. We have made our best efforts to incorporate all of these requested changes where possible and discuss all of the findings of our study.

**Comments from Reviewer #4**

Line 268 , “Within the new case group, detection of RLEP was 87.3% (62/71) in MB cases and 68.8% (11/16) in PB cases. When cases were subdivided according to Ridley-Jopling classification for the different forms across the disease spectrum, RLEP amplification was positive in 57.1% (3/7) for the indeterminate form, 80% (4/5) for TT, 87.5% (42/48) for BT, 84.2% (16/19) for BB and 100% for BL and LL (4/4). In addition, four cases of primary neural form (PNL) were diagnosed, and all were positive (100%, 4/4).”
Similar results can be documented for anti-PGL-I.

We have added the anti-PGL-I results for each form across the disease spectrum for the new case group. “Within the new case group, detection of a positive anti-PGL-I titer was 68.8% (42/61) in MB cases and 37.5% (6/16) in PB cases. When cases were subdivided according to Ridley-Jopling classification for the different forms across the disease spectrum, anti-PGL-I was positive in 57% (4/7) for the indeterminate form, 20% (1/5) for TT, 70.8% (34/48) for BT, 31.6% (6/19) for BB and 50% for BL and LL (2/4), respectively. In addition, four cases of primary neural form (PNL) were diagnosed, and 25% (1/4) were positive.”

comment no 9) The author needs to mention about the age groups of subjects and among the cases positive for any test in all groups, was there any child case?

The total population evaluated is made up of 466 individuals, of which 92/466 (19.7%) were under 15 yo. We have added this to the Results section. The median age and range for each group is found in Table 3.

However, we did not find the above results mentioned in the revised manuscript. Also, findings on the number of children in new, treated and HHC group, will give information about the transmission.

Thank you for pointing out this omission. Of the 87 new cases, 38/87 (43.7%) were in children under 15 years old, while 3/52 (5.8%) of the treated group, 51/296 (17.2%) in the HHC group and 0/31 in the HEC group were children, respectively. We have added the information on the number of children in each of the four groups in the beginning of the Results section.

Line 337 - For this reason, we have been using less invasive methods, namely taking samples of blood and earlobe SSS. (In response to our comment no 5) If skin smear BI available in Index case was there a correlation between high BI positivity and HHC positive.
Since most of the work was done in the field, the BI of index cases was not assessed.
However, we always do a bacilloscopy on leprosy cases attended at the UREMC reference center, resulting in a high correlation between our clinical definition and the BI.
We suggest that to obtain the BI, SSS from all earlobe, forehead and active lesion can be taken on slide, heat fixed, stained by ZNCF and quantified under oil immersion. There is no need of taking invasive punch biopsy.

In response to the question about the BI, we only obtained samples of earlobe SSS and serum to perform laboratory PCR for M. leprae DNA and anti-PGL-I titer by ELISA. We agree with the reviewer’s statement that for a proper assessment of the BI at a leprosy reference laboratory, generally six SSS samples are taken from several sites for making smears on slides, heat fixed and stained by Z-N. In the field, this was not feasible.
