# Supplementary material for: Latent leprosy infection identified by dual RLEP and anti-PGL-I positivity: Implications for new control strategies
Source: PLoS One. 2021 May 13;16(5):e0251631. doi: 10.1371/journal.pone.0251631 (PMC8118453; doi:10.1371/journal.pone.0251631)
Supplement: Attachment — Submitted filename: PONE-D-20-1981 R2 Review.docx [file pone.0251631.s010.docx]

Overall, PONE-D-20-19814R2 is better written compared to PONE-D-20-1981. Some of my comments were addressed in the review however; I still have a few more comments.

Introduction

The introduction does clearly explain the problem, but it could be summarized. The flow is good till line 109, however after that what was done should have been summarized.

Line 145-to Line 154 is actually Methods and Materials and the results in the introduction section. Despite the authors attempt to highlight that they are giving a rationale for the investigation. They actually stated the study design and what they found. If they were tempting to give the hypothesis, there is need for re-wording

Methods

The methods and materials have improved however the study cannot be reproduced.

It may be important to state the sampling methods used to get the evaluation areas. The authors do not that it was divided into Rural and Urban. But there is no other mention of any sampling technique and why it was chosen.

It may be important to find out how the authors arrived at 466 individuals with 87 newly infected, 52 former patients, 296 household contacts and 31 healthy endemic controls. Was this also random or it was the available data?

There is completely no mention of how the data was collected or stored prior to being analyzed

Results

Line 255 to 256, in the results section we expect to get the results got from the use of the t-test not what statistical test was used.

Discussion

This may seem like a contradiction on my last review, but I think there is need summarize the key finding of the study and discuss only those.
