# Supplementary material for: Latent leprosy infection identified by dual RLEP and anti-PGL-I positivity: Implications for new control strategies
Source: PLoS One. 2021 May 13;16(5):e0251631. doi: 10.1371/journal.pone.0251631 (PMC8118453; doi:10.1371/journal.pone.0251631)
Supplement: Attachment — Submitted filename: Response to Reviewers.doc [file pone.0251631.s011.doc]

We again thank the reviewers very much for their additional valuable input and suggestions which were very helpful. We have endeavored to answer all of the questions and make the changes to the manuscript based on these suggestions wherever possible. Our point-by-point responses are in blue after each reviewer comment and describe the changes made to the manuscript in response to their comments. In addition, we have numbered the remaining comments from Reviewer #3 and indicated specifically where the changes were made in the “Manuscript with Track Changes” to highlight the responses, with added Comments in the margins addressing each of the comments from the reviewer. We hope that this third revised version of this manuscript addresses the remaining issues.

PONE-D-20-19814 R2

Title: Latent leprosy infection identified by dual RLEP and anti-PGL-I positivity: Implications for new control strategies.

PLOS ONE

Thank you for submitting your revised manuscript " Latent leprosy infection identified by dual RLEP and anti-PGL-I positivity: Implications for new control strategies" to PLOS ONE. Peer review of your manuscript is now completed. Based on these reports and my own assessment as Editor, I am pleased to inform you that it is potentially acceptable for publication in PLOS ONE once you have carried out some essential revisions suggested by a reviewer. Currently, reviewer-3 has raised few genuine concerns which need to be addressed. If you can't address all the issues, I recommend discussing those points in the discussion part of the manuscript or state as a limitation of the study. Please submit your revised manuscript by 03 April 2021.

**Comments from Reviewer #4**

It is observed that the comments made earlier in the review have been addressed satisfactorily. The comments made have been responded to and have been also incorporated in the manuscript. As the comments have been addressed and incorporated in the manuscript the issues related have been clear and it therefore helps to relate and convey the objectives and purpose of the study and the results arising out of the study which are very important and have epidemiological implications.

We thank the reviewer very much for his kind remarks about our work and we are pleased that we have addressed all previous comments and made changes that are acceptable in the manuscript.

**Comments from Reviewer #3**

Overall, PONE-D-20-19814R2 is better written compared to PONE-D-20-1981. Some of my comments were addressed in the review however; I still have a few more comments.

Comment 1: The introduction does clearly explain the problem, but it could be summarized. The flow is good till line 109, however after that what was done should have been summarized.

In the previous revised version, we significantly cut, revised and summarized the Introduction. We eliminated nine sentences about the Dr. Marcelo Candia Reference Unit in Sanitary Dermatology (UREMC) as requested. In the first paragraph (lines 82-98), we included a summary of the number of cases of leprosy worldwide with particular mention of the situation in Brazil, which is the only country in the world that has not met the WHO goal of <1 new case per 10,000 population. Leprosy is a particular concern in the northern state of Pará, where this study occurred, where it is considered hyperendemic (>4 new cases per 10,000). In the second paragraph (lines 100-109), we summarize that there is no laboratory test capable of detecting all clinical forms of leprosy and that it is ideally diagnosed by well trained dermatologists and leprosy clinicians based on clinical signs and symptoms, but because this is often not done, it leads to delays in the diagnosis. Developing additional tests that can detect early or asymptomatic leprosy is a research priority. In the third paragraph (lines 111-135), we summarize the classic clinical signs and symptoms of leprosy that guide the diagnosis on physical examination. We also summarized the five Ridley-Jopling forms and the treatment required for MB and PB disease. Adjunct tests used to aid in determining length of treatment or confirm the diagnosis include the anti-PGL-I ELISA and detection of M. leprae DNA by PCR, which were summarized. The final paragraph (lines 137-142) summarizes how we combined both of these tests in a cross-section of leprosy patients, healthy household contacts and healthy endemic controls from 7 different regions in the state of Pará to identify those with one or both of these biomarkers of infection.

Comment 2: Line 145-to Line 154 is actually Methods and Materials and the results in the introduction section. Despite the authors attempt to highlight that they are giving a rationale for the investigation. They actually stated the study design and what they found. If they were tempting to give the hypothesis, there is need for re-wording

This comment was responded to in our previous revision where this sentence was crossed out and eliminated from the end of this paragraph, so lines 145-154 no longer exist. The sentence that was eliminated in the previous revision is as follows:
“We show that the majority of newly diagnosed leprosy cases with clinical symptoms are positive for both of these biomarkers of infection suggesting the possibility that household contacts who are also double positive may have latent disease and should be carefully monitored through follow-up examinations.”

Comment 3: It may be important to state the sampling methods used to get the evaluation areas. But there is no other mention of any sampling technique and why it was chosen.

Dr. Claudio Salgado has been performing active leprosy surveillance in the state of Pará since 2009 and has performed these active surveillance activities in over 35 different cities in the state, some of them visited multiple times for follow-up studies of household contacts that we have published. We generally perform active field surveillance at 3-4 cities per year, examining and obtaining serum and SSS from between 100-500 individuals at each location during a 5-day period. Our previous published studies have shown that on average of 4% of schoolchildren and 8% of household contacts can be diagnosed with clinical signs and symptoms of leprosy in this state, a number that is hundreds of times the new case detection rate for Brazil which is based almost exclusively on passive detection of cases. As mentioned in “Study area”, the municipalities selected were “To include a broad representation of individuals from cities of various sizes, we selected seven municipalities that came from the six mesoregions in this state.” These sites provided a diverse set of municipalities and study subjects from very small towns with less than 12,000 population to the capital city with a population of 2.3M. All of the cities visited were selected for active field surveillance based on the logistics of getting there safely and our ability to drive to these cities as many places in Brazil are inaccessible due to the lack of decent roads. One of the smallest towns surveilled, Senador José Porfirio, required us driving over 15 hours from Belém on the TransAmazon Highway that is still only partly paved. Another city, Breves, can only be reached by an overnight boat trip in which the vehicles with supplies used by us to get to the study subjects were also loaded onto the boat. The city of Santarém was reached by our team by commercial plane. Once there, some of the support crew who live there assisted in transporting our team to visit the study subjects. Thus, the logistics of chosing the sites, visiting patients and household contacts and obtaining the biological specimens for analysis can be very complicated, requiring permission and assistance from local health ministries and personnel from the basic health units and planning for these active surveillance trips takes several months in advance.

Comment 4: The authors do not that it was divided into Rural and Urban.

We apologize for this but it is unclear what the question is. According to Table 3, the number of subjects from all four groups living in the urban area was between 80.8% to 100%. We consider people living in a rural area to live within the city limit boundaries but visiting them may have required driving on a dirt road in the rain forest for an hour or more, so definitely rural. Those living in rural areas for each group ranged from 19.2% to 0%, so subjects from rural areas were in the minority. We have highlighted the column showing this data in Table 3.

Comment 5: It may be important to find out how the authors arrived at 466 individuals with 87 newly infected, 52 former patients, 296 household contacts and 31 healthy endemic controls. Was this also random or it was the available data?

This question was answered in the previous revision. The first three groups (new patients, treated patients and household contacts) were from a set of available data. The data from 31 healthy endemic controls were added in response to a request from a reviewer that we needed to add this control group when the manuscript was submitted to PLoS Neglected Tropical Diseases the first time in June, 2019. The numbers of individuals from each group for each of the seven cities visited can be found in Table 1.

Comment 6: There is completely no mention of how the data was collected or stored prior to being analyzed

We thought we had addressed this question in the previous revision. We assume that you mean “biological samples” for “data”. We have added two sentences regarding the processing and storage of the serum and earlobe SSS in “Study Design and Methods”. “Blood was processed by centrifugation at each site to yield serum that was frozen and transported to the laboratory for analysis by ELISA. Earlobe SSS samples were placed in microcentrifuge tubes containing 70% ethanol and transported to the laboratory for DNA extraction and analysis by PCR.” We have indicated in the text where these sentences for this change are located.

Comment 7: Line 255 to 256, in the results section we expect to get the results got from the use of the t-test not what statistical test was used.

We thought we had addressed this comment in the previous revision. We changed this sentence to state that the p values were calculated to show significant differences between groups. We have now indicated the location of this change in the text for clarity.

Comment 8: This may seem like a contradiction on my last review, but I think there is need summarize the key finding of the study and discuss only those.

We have made our best efforts to incorporate all of the requested changes from each of the original five reviewers where possible and discuss and summarize all of the findings and limitations of our study. For example, the paragraph from lines 411-427 discussing the interpretation of the four different combinations of anti-PGL-I and RLEP was inserted at the request of Reviewer 5 in one of the earlier revisions. Table 3 and the paragraph from lines 304-318 in the Results and 429-447 from the Discussion discusses the demographic information and possible risk of being diagnosed with leprosy associated with income levels, education level, food insecurity, lack of clean drinking water, overcrowding and other factors. These various factors and their possible involvement in disease progression resulted from previous comments from Reviwer 3 to include this data. Although not originally included in our study, we feel that this data does illustrate that those in the patient, treated patient and household contact groups are likely at greater risk for leprosy and negatively impacted by poverty, lack of sufficient food intake leading to hunger, high density living, lack of clean drinking water possibly leading to co-infections that could lead to a compromised immune system and lower levels of education than the healthy endemic control group. We thank the reviewer for asking us to include this information.
