# Supplementary material for: Latent leprosy infection identified by dual RLEP and anti-PGL-I positivity: Implications for new control strategies
Source: PLoS One. 2021 May 13;16(5):e0251631. doi: 10.1371/journal.pone.0251631 (PMC8118453; doi:10.1371/journal.pone.0251631)
Supplement: Attachment — Submitted filename: Response to Reviewers.doc [file pone.0251631.s012.doc]

We again thank the reviewers and the editor very much for their valuable input and suggestions which were very helpful. Based on the suggestion from the editor, we have added a paragraph “Limitations of the study” after the Discussion. We hope that this fourth revised version of this manuscript addresses the remaining issues.

PONE-D-20-19814 R4

Title: Latent leprosy infection identified by dual RLEP and anti-PGL-I positivity: Implications for new control strategies.

PLOS ONE

Thank you for submitting your revised Manuscript to PLOS ONE. It has been suggested to add the "Limitations of the Study" to the manuscript after the discussion part in the previous editorial decision. However, I could not see it in the revised submission. If you have added it, please highlight it in colored text, and if you are not willing to add it, please respond with reasons in the letter.  Now we are returning your manuscript and invite you to submit a revised version that addresses the points raised during the editorial process.

Please submit your revised manuscript by 30 April 2020.

**Response to request from the editor**

Thank you for your suggestion. We have added the section “Limitations of the Study” at the end of the Discussion as follows:

The diagnosis of leprosy is still based on the detection of classic signs and symptoms of skin lesions with loss of sensation, nerve damage with loss of function, swelling or pain and visible deformity as detected by well-trained clinicians or health care personnel since *M. leprae* cannot be cultivated. Despite the use of adjunct laboratory tests to detect acid fast bacilli in skin smears or *M. leprae* DNA by PCR, these tests are not always available in resource constrained settings. The anti-PGL-I assay, although easy to perform, only indicates prior infection by the bacillus and a positive test by itself does not trigger the administration of MDT since the majority of positive individuals will not progress to clinical disease. Although we have shown that the majority of newly diagnosed leprosy cases with clinical symptoms are positive for both anti-PGL-I and *M. leprae* DNA by PCR, whether household contacts who are also double positive have latent disease and are at the highest risk of succumbing to disease can only be determined in future long term longitudinal follow-up studies.
